# Experimental Researches on the Post Mortem Contractility of the Muscles, with Observations on the Reflex Theory

**Published:** 1846-08

**Authors:** 


### ECLECTIC DEPARTMENT.

*Experimental Researches on the Post Mortem Contractility of the Muscles, with observations on the reflex theory, by* BENNET DOWLER, M. D. *of New-Orleans, Louisiana.*

This is the title of an article in the New-York Journal of Medicine for May, 1846. The author, Dr. Dowler, is well-known to the profession from a series of interesting observations made by him on the temperature of the human body after death, or, as he styles it, post-mortem fever. He now submits some highly curious and interesting experiments upon the contractility of the muscles after death, the results appearing to conflict with the new views which recognise, in addition to the cerebro-spinal system of nerves, a system having automatic functions independently of these — known commonly as the reflex, true spinal, or excito-motary system of Marshall Hall, Grainger, Muller, and others. The distinguishing traits of this theory are given by Dr. Dowler in two quotations, one from the Medico-Chirurgical Review, and the other from the Am. Journal of Medical Sciences. In order to refresh the recollection of our readers respecting the views which Dr. D's. observations tend to invalidate, we transfer the quotations just referred to. The following is from the Medico-Chirurgical Review :

"It is at length admitted on all hands that here (in the spinal cord) impressions do pass, and necessarily, from the afferent to the efferent fibres.' 'The impression (necessary to muscular motion) is conducted to the spinal marrow, is thence reflected, and again reaches either the part on which the impression took place, or some other part remote from it, where the muscular contractions now take place'—'*in an arched and reflected course, requiring that the connection with the spinal marrow be uninjured*'—'a throwing back of the nervous principle into the spinal marrow, without at the same time admitting a process similar to sensation.' This process is also compared to the '*reflection of light.*' "

The following is from a review of Dr. Dr. Hall's writings in the American Journal:

"Five circumstances are required in every instance of an excito-motary act; an excitant, *an excitor nerve, continuous to the nervous centre; the integrity of that centre: a motor nerve, continuous to the muscle to be excited into contraction;* and that muscle to be endowed with perfect irritability. *If any part of the arc be interupted, the phenomena cease instantly.*" "There can be no better test of the value of these views than the contest which has arisen, not with regard to their truth, but their originality"—"they have gained ground in despite of all opposition." *All action is the result of stimuli to the nerves which proceed to the spinal marrow, pursuing a reflex course,*"—"*By two lines.*"

The experiments conducted by Hall, from which these deductions were made by him, are, probably, familiar. They are are concisely stated by Dr. Dowler, using the language of Dr. Hall, as follows:

"Dr. M. Hall, in his work on the *Nervous System*, says that he "divided the spinal marrow of a frog below the occiput—on pinching its toes with the forceps, the extremities moved," but "on destroying the spinal marrow with a probe, they were, on pinching, motionless." "The limbs of an animal, or a part of an animal, separated from the influence of the cerebrum, become relaxed on destroying the spinal marrow." "On withdrawing gently the spinal marrow of a decapitated turtle, all motion ceased. The limbs were no longer obedient to stimuli, became perfectly flaccid, having lost all their resilience. The sphincter lost its circular form and contracted state, and became lax, flaccid and shapeless." "*The presence of the Medulla Oblongata and Spinalis is necessary to the contractile function.*" "To irritate the spinal nerves will produce contractions the very type of tetanus, leaving no doubt of what particular part of the nervous system is affected in that disease." Finally, he concludes, that these "effects have not been hitherto suspected by Physiologists." "

It is well known that the *subjects* of the experiments upon which the doctrines of the reflex school are based, were taken chiefly from the Batracian class of animals. Dr. D. objects to making the results of experiments upon these animals applicable in all respects to the human organism. He says,

"It should never be forgotten that experiments on the inferior animals, as frogs and turtles, are inconclusive in establishing the complicated physiology of man. An earth-worm may be cut into several pieces, each of which becomes a perfect animal. The more the *hydræ* are subdivided, the more is their number increased without loss of vitality. Could we deduce a vital or reflex theory from such experiments applicable to man? Were capital punishment changed from hanging to vivisection of the spinal marrow, this would but poorly illustrate the physiology of a healthy man, or the pathology of a sick one, yet one such experiment would be worth a thousand upon frogs."

And again, with reference to some conclusions of M. Matteucci, founded on experiments with frogs:

"At best, this is only a piece of frog physiology, that may not have anything to do with human physiology. Nothing in the history of fashion can be more extraordinary than the *penchant* of physiologists for that ugly, amphibious animal, the frog, so unlike man in its structure, respiration, circulation, temperature and maladies; yet it seems to be the type for all things concerning human beings, not excepting inflammation and muscular contraction. Why not include gout, consumption, cholera, and mesmerism? The sacred Ibis was never so honored by the Egyptians as is this ranine amphibium, by the *savans* of our day. As this great idol of experimenters is capable of living at the bottom of a frozen pond or lake during half the year, and may be skinned, nay, decapitated without losing the power of locomotion for a considerable time; it must be admitted that its vital versatility is well adapted to prove almost anything, especially when aided by a few assumptions and manipulations of a galvano-neurological character?"

The coarse, cruel and fallacious experiments practised, more especially by some of the French Physiologists, are justly repudiated.

Sir Charles Bell in his work on the nervous system, says:

"Cold blooded animals will live without the brain. Birds, whose heads were cut off, Le Gallois says, walked, seemed to feel pain, and moved their feet towards the part. Flourens goes further, since he says that a bird deprived of the cerebral lobes dressed its feathers, and ran and leaped." —P.25.

Such experiments, including many of the author's prickings and cuttings of the spinal marrow, can have no satisfactory application to man. The same may be said of M. Magendie's experiments.* He wishes to know the functions of certain nerves; he divides them at their origin, plunging a sharp instrument through both sides of the skull at the base of the brain, cutting up the cerebral matter, and perhaps several other nerves, in order to divide the fifth; nay, more, he removes both hemispheres of the brain from a duck. This animal, for eight days after, appeared to do very well, and as he assures us, possessed the sense of smell! Now the question is, does man exhibit such phenomena after the removal of the brain, or the division of its nerves within the cranium, or the dissection of the anterior and posterior roots of the spinal nerves?"

"As illustrative of the nature of these experiments, I offer the testimony of an abler champion of the reflex school:—"The experiments" says the editor of the 'Medico Chirurgical Review' "which have been devised and practised for ascertaining the functions of the nerves, are the most cruel that can be imagined, while the results thereby obtained have frequently been unconvincing or uncertain." This language, repeated from year to year, gathers strength in the last number of that journal (Oct. 1845), in which the editor denounces vivisections, and "protests," to use his own words, "against those barbarous experiments on living animals, the performance of which is so disgraceful, upon the whole, to the medical profession. And what good have they done?—literally none. We verily believe that there would have been much more rational views upon many disputed questions in physiology, if observation had been exclusively confined to noticing the natural and spontaneous phenomena of life in health and disease, and diligently examining after death the effect which disease leaves behind. Shall we believe that any conclusion worth knowing can be drawn from cutting a poor animal's heart out of his body? Away with such butcher-like philosophy!" "

Irritating the roots and trunks of nerves, and passing through them the electric current, have been much employed to determine their offices and relations. Dr. Dowler thinks these methods are but of little avail, inasmuch as, by the former, in the recently dead human snbject, few, or no sensible effects can be produced; and in the latter, the agent used is of such power,

* English critics assign to this distinguished physiologist. the second seat of honor in this department of discovery.

that its effects are not to be received as evidences of natural functions of living organs.

We come now to the method pursued by Dr. Dowler, which certainly has the merit of simplicity; but it is not less worthy of attention and confidence on this account. The method consists in making percussion with the hand, or some solid body, directly upon the muscle, (through the integument) or muscles, whose contractility It is desired to affect. He was led to the discovery of this method by accident. The following is his account of the circumstances connected with the first experiment:

"Whether these experiments be of any value or not, I trust that candid men will not estimate them the less when I say that they were made, not to order, but rather incidentally, while engaged in pathological inquiries which occupied my attention almost entirely. For nearly two years after I first noticed post-mortem muscular contractility, I seldom thought of its special application to the reflex theory. I viewed it as a curious, and, so far as I knew, an original observation. During an attempt to produce contusions on the recently dead body, I happened to select a spot over the middle of the biceps; a blow there (the arm having been extended on the floor) caused the subject to slap his hand against his face with much force. I was almost as much surprised as was a black man that I hired to aid me in a private dissection, in St. John the Baptist street, in 1842, of the body of a gentleman of much travel, who died of remittent fever. Previous to this dissection, which was two hours after death, I struck the arm with the inferior edge of my extended hand. The subject contracted his arm, carrying his hand to his breast. My aid looked to the door, which had been closed beforehand, and begged to be let out without delay.

It was, therefore, very recently that I began to regard contraction thus excited in its most elementary character, as an important point of departure in the analysis of some of the most interesting problems in physiology, &c. It cannot be repeated too often, that the simplicity of a method is a great recommendation, while complexity gives rise to suspicion when an explanation is offered, and at the same time, increases the chances of error. Professor Liebig has said with force, that "if a method of investigation bear in any degree the stamp of perfection, the result may always be given in a few words; but these few words are eternal truths."

With regard to the originality of this method we will allow the writer to speak for himself:

"Has muscular contraction, by the percussional method, ever been pointed out by the authors? Have they noted contractions without any shortening of the muscle, though accompanied with an enormous augmentation of volume and density at the points of contact? Have they ascertained, fixed, and made known the laws of muscular action, as developed by this most definite and simple of all methods, divested, as it is, of volition and all psychological phenomena? Have they described a case of complete motion of the whole limb, not only including flexion, but extension, pronation, supination,—in a word, all the elementary, physiological actions, simulating the voluntary movements, though less varied and more slow; but, more than all, have they illustrated the great law of contractile periodicity, excited *per se*, in a manner very different from the unmeaning convulsions of electrical action,—from the unmeaning action of a "scratch or prick with a sharp instrument?"—I say unmeaning, because no definite actions are produced resembling those called physiological. The use of the phrase "sharp instrument," from Haller's time to the present, speaks volumes in favor of my position,—for a dull instrument, as a shingle, cane, the flat side of a hatchet, or the operator's hand, is infinitely better, because it moves the entire limb, whereas, a scratch causes nothing of the kind, giving rise to nothing but a delicate ridge or line, like a thread, in any direction across a muscle, diagonally, triangularly, or in curves, as the case may be.

It is not, then, the silence of authors upon which the greatest reliance is to be placed, in claiming for this method of investigating muscular contraction, something of originality. All writers, so far as recollected, especially writers on Medical Jurisprudence, in treating of the signs of the certainty of death, rely much on muscular contraction, or as I should say, on its absence, as the most indubitable sort of proof. During the present year, M. Cadet De Grassicourt, a French writer, proposed, as the best method of ascertaining the reality of the supposed death, to disect down to the muscles and prick them— a common recommendation in such cases,—"As if a man be dissected to see what part was dissaffected," and this, perhaps during his lifetime! (I regret that I cannot stop here to examine into the fallacy of this TEST, which would go to prove the absence of real death, or the possibility of resuscitation after the removal of the brain, spinal marrow, the viscera of the chest and abdomen, and the amputation of the limbs at from six to eight or fourteen hours after death!) Now, as percussion is the only perfect and safe method of exciting contractions, I cannot suppose that there is a single medical man, who, knowing this, would lay bare, by a surgical operation, the muscles of a person supposed to be possibly alive, in order to apply a test of little or no value, when one of the greatest value could be commanded by a simple blow of the inferior edge of the operator's extended hand, insufficient in force to injure a living person. Here, as in law, the rule should be that the best evidence ought to be procured.

During the last ten years, many French authors, candidates for the prize founded by professor Manni, of the University of Rome, to be awarded by the French Academy of Sciences, have written ably upon the signs of apparent and absolute death, with a view to prevent premature interment, etc- In this question, which is still open and undecided, much importance is constantly attached to contractility, or rather to its absence, requiring a dissection in order to expose the muscles for this test! It is, in fact, a fundamental point which is made paramount to all other signs of death anterior to putrefaction. The celebrated "Nysten recommends this as a certain criterion of death—to lay bare the muscle and prick it, or apply the galvanic or electric fluid." (*Prof. Guy's Med. Juris., by Prof. Lee,* 1845, *p.* 372, *et seq.**) These, though negative proofs, are nevertheless

* This work contains an enumeration sf the signs of death, which, with the other topics treated of, are more clearly and compendiously stated, than in any work I have seen from the foreign press. Dr. T. E. Beatty (*Cyc. Prac. Med.*) maintains that "Galvanism is the best test of the reality of death; for, if the muscular fibre do not obey it, there can be no doubt that vitality is extinguished." Galvanic contraction is no proof of the presence of life, or of the possibility of resuscitation.

sufficient to show that the method I have adopted to excite muscular contraction is wholly unknown, or absurdly rejected. Why should a very simple matter (I ask once more) be thus complicated by galvanic experiments, dissections, and reflex logic, seeing that a blow direct with the hand will produce results more definite, more like the acts of vitality and volition? If an arm, from six to fourteen hours after death—hours after the dissection of the body, and after being severed from the trunk at the shoulder-joint, contract with the utmost precision, how can gentlemen say "that the anterior columns of the spinal cord possess an exclusive motor function"? Whatever agency these roots may have when galvanized, they are incomparably inferior to the motor power of the muscle when excited by the hand, the hatchet, or a cane. A hand, or a hatchet, or a cane-theory, ought to have the preference over the reflex theory, as the former is not only the most simple, but the most scientific, affording a nearer approximation to the known physiological functions of man. Of this the sequel will afford evidence—evidence which was scarcely sought for, but which came of itself, incidently—evidence, simple, plain, and direct, and not obtained under the tension of any theory whatever."

Dr. D. proceeds to adduce cases in which experiments were made and the phenomena noted. He selects twenty cases illustrative of the *general phenomena of post-mortem contractility.* A few of these will suffice to give our readers a general idea of their character. We omit in our quotations the nativity of the subjects, which seems to us irrelevant, and not in good taste.

"CASE I.—O. D., aged 26. From fifteen minutes to several hours after death, raised his fore-arm to the perpendicular as often as its flexors were struck; the motion was slow, the ratio of contraction and relaxation appeared uniform, occupying about half a minute."

"CASE II.—E. M., aged 37. At thirty minutes after death, afforded similar results. The experiments were often repeated, at prolonged periods; the contractions and relaxations were supposed to occupy about 30 seconds each. The flexion ceased at the perpendicular *in* every instance. When the experiments ceased, the muscular force continued at its maximum, having lost nothing."

"CASE IX.—Miss S., aged 26. An hour after death presented slight rigidity of the arm; one was extended; a blow caused it to rise quickly to the perpendicular, from which it slowly returned, requiring many seconds, perhaps a minute or two. Three similar operations destroyed the contractility. The fingers soon became stiff, then the arms, next the legs, but the neck was limber for three hours. The heat generally 107°, descended in the axilla, at four hours after death, to 103½°."

"CASE XVII.—R. C., aged 25. In two hours after death, when the arm was extended to an angle of 45° from the trunk, and was struck with my hand, or still better, with the side of the hatchet, carried his hand to his epigastrium; but when the arm was extended upon the floor, so as to form a right angle with the body, he slapped himself upon the mouth and nose. The contractility began to decline in the third hour, and by the fourth hour all motions of the limbs ceased, though the pectoral muscle assumed the ridgy or lumpy form when percussed. An hour after death the thigh was moderately contractile. The leg hung down near the floor, its flexors, after being struck, drew up the heel against the buttock. Heat for seven hours, from 111° to 102°. Five hours after death contractility ceased, and rigidity prevailed."

Case XX.—*Remittent.*—Mr. S., aged 45. Dead two hours; legs becoming rigid; struck the flexors of the arm with the inferior edge of my hand; the cadaver raised his arm with a regular, slow movement, placing his hand upon his breast: as soon as the muscles relaxed, he carried his arm back, extending the same. The experiment was repeated three or four times, when the arm fell back exhausted. The blows were now made with a piece of wood. The biceps gathered up into a lump, at the place where the blow was given, but failed to move the fore-arm."

*Cases of Post-Mortem Contraction without Shortening.*—On this subject the author remarks as follows:

"It is natural to every muscle, says Haller, to shorten itself, by retracting its extremities towards its belly or middle; when in action becoming shorter and thicker (cccci). Whether this doctrine be true of galvanic muscular contraction, I do not know, but I am certain that it does not apply to post-mortem contraction,—because it often happens that both the fore-arm and elbow-joint are stiff and immovable, while the biceps may be powerfully contractile, its belly swelling up into a hard lump; relaxing and contracting repeatedly, and unavailing, owing to the rigidity at and below its insertion in the arm. Here, it is evident that the indurated swelling is not owing to the approximation of the two extremities of the muscle, for they are fixed. The increase of volume seems to be owing to an expansion with rigidity among its elementary fibres; a zigzag oscillatory motion upon the summits of the contracting masses is visible to the naked eye. Much evidence might be produced besides the following:"

He gives several cases in illustration of the above fact. A single one will answer our present purpose.

Case XXII.—R. P. aged 24. Dead ten hours; fore-arm rigid; the biceps contracted strongly, but the rigidity in and below the elbow-joint prevented flexion. Blows with the knuckles over the pectoral muscles caused them to heave up into knotty masses.

Sometimes, post-mortem *rigidity sets in duringthe paroxysm of contraction.* producing a very singular phenomenon, a hard mass, which, continuing for hours, feels like bone, I have known this to be mistaken for a fracture badly set, or for a bony tumour."

*Cases illustrative of the increase, declination, and subsequent resuscitation of Post-Mortem Contractility.*

Case XXV.—A. G. G., aged 30. In a few minutes after death, presented but feeble contractions, which, in half an hour ceased for a time. But soon after the contractile function returned to the same arm with much force, but after repeated blows it was exhausted a second time. Again, after a similar interval, it returned with a like force a third time. In three hours the rigidity, beginning in the neck, extended itself to most parts of the *cadaver*. The blows were found to have caused well-marked contusions, cellular ecchymoses, &c."

Three other cases of a similar character are given. It should perhaps be stated, (as it is by the writer,) that nearly all the subjects in the preceding, and following experiments were dead from yellow fever.

*The destruction of the contractile function in one arm does not affect the other; this rule has no exceptions.*—This postulate is sustained by experiments, of which one is adduced, as follows:

"CASE XXIX.—S. F., a Cincinnatian, aged 54. Dead half an hour when the experiments began. The right arm was struck with the flat side of the hatchet, the hand was carried to the ear; the second time to the mammary region; a third to to the perpendicular; a fourth caused but a slight motion, and two more, more severely laid on, completely killed the contractility, leaving the print of the instrument as upon dough. In an hour after, the left arm was found to be contractile as the right had been, if not more so. The thigh, two hours after death, and an hour after the removal of the viscera, gave nearly 107°. Other cases illustrative of this point are deemed superfluous."

That the contractility thus found to be inherent in the muscles after death has no connection with the presence of blood in the muscular tissue, is shown by several experiments made upon subjects who had died of hemorrhage, and, also, as will be mentioned presently, in portions of the body separated from the trunk, their vessels divided, and consequently drained of their contents. Dr. D., therefore, deduces that the following statement of Sir Charles Bell, in his work on the hand, requires, in the dead subject at least, some modification:—

"The muscular tissue has a living endowment, a power of contraction and relaxation, which is supplied by the circulation of the blood, the source of all vital power."

It was a subject of enquiry with Dr. D. whether the presence and continuance of P. M. contractility had any relation with the presence and continuance of P. M. coloricity. He presents a collection of cases in which observations, both of heat and contractility, were simultaneously made. These we need not quote, but simply give his conclusion, which is, that "the continuance of, or rather the degree in which post mortem heat is evolved, bears no proportion to the intensity of post mortem contraction."

The preceding experiments, although highly curious and valuable, are yet not necessarily opposed to the reflex theory. If this theory does not afford the readiest explanation, it is not inconsistent with the facts to suppose that in all the instances the effects of the percussion were exerted through the incident and motor nerves, thus involving the transmission of an influence, first, to the spinal cord, and, next, reflected to the muscle which contracts upon the application of the percussion to it. This would seem to be a far-fetched rationale, if the reflex theory of muscular contractility did not exist; but with the views now in vogue, it would appear perhaps, most probable. This explanation, however, is hardly available in the cases which follow; which, certainly, in so far as the results of these experiments go, are entirely incompatible with a reflex current through the spinal marrow.

"CASE XLI.—A. M., aged 32. More than four hours after death, and an hour after dissection, possessed the contractile function in a high degree. An arm was amputated so as to include the scapula and its muscles; after which, blows were repeatedly directed upon the flexors of the fore-arm, which was as often elevated to the pependicular; one of the inferior extremities, an hour after amputation at the hip-joint, being generally rigid, presented, nevertheless, contractility in the form of massive ridges at the places of contact from blows."

"CASE XLII.—P. L., aged 20. Dead four hours before the experiments were begun. Both fore-arms raised themselves to the perpendicular several times. The left arm was amputated so as to include the scapula and its muscles. Twenty ounces of blood by estimation were rapidly discharged by the operation. The temperature had ranged for more than four hours in different regions, at from 34° to 33° of Reaumur (nearly 109° to 106½° of Fah.). The arm was more contractile after than before its separation from the spinal marrow, rising after each blow without any loss of motor force as long as observed."

"*Cases showing that Amputation of the Shoulder, and the subsequent Division of all the Nerves, Vessels and Muscles of the Arm, except the perpendicular Flexors of the Fore-arm, do not in the least impair the contractile function either in force or duration:—*

CASE XLIII. J. G. aged 32. Dead three hours; dissected one hour; viscera all removed from the body; the right arm was extended, and slapped with a piece of shingle, the force being scarcely sufficient to injure a living person. This blow was repeated at intervals five or six times; each time the man struck his hand against his shoulder with great celerity and force. A heavy hammer was then tied in the palm of the hand, but even this failed to counteract the muscular force; he struck himself heavily against the breast. A hatchet estimated at 2½ to 3 lbs, was substituted for the hammer; with this he struck the breast several times, when the contractility suddenly ceased. One hour after this, and more than four after death, both arms, including the scapulary muscles, were amputated discharging enormous quantities of blood—hot blood—for the post-mortem temperature had been ranging from 36° to 34° of Reaumur (or 113° to 109° of Fah); the left arm was extended on the table, resting on the hand and on the olecranon, on the angle of the scapula and the sternal end of the clavicle, presenting the deltoid uppermost. The most active contractions followed every blow, the arm twisted about like a wounded snake, the muscles of the scupula were convulsed. The shoulder having lost its *point d'appui* upon the trunk, the whole arm some times turned once, so that the deltoid rested upon the table. Gradually, as in the former case, the contractility began to decline. I now severed all the muscles of the arm, in its lower third, except the biceps and brachialis, including all the accessible nerves and vessels, but I found the contractility as active as before. It was, of course, moderate from the previous exhaustion. The amputation at the shoulder, the cutting of all muscles, &c., but the flexors, had not impaired the contractile function in the least. The amputated limbs remained very warm about three hours."

It has been already remarked that the subjects of these experiments were generally the bodies of those who had died with yellow fever. Dr. D. has found that these subjects exhibit more frequent and enduring cases of contractility than those dead with other forms of fever. This, however, is not due solely to the rapid march of the disease toward a fatal termination, since, among yellow fever subjects, in those cases in which death occurred most quickly, it was not found that contractility was more uniformly present, or was presented in a greater degree.

Dr. D's experiments determine for the duration of contractile power in the muscles after death, a considerably longer period than has been given to it by other writers. Prof. Guy, for example, limits its duration, in extreme cases, to two hours (Med. Jurisprudence). Prof. Dunglison, to two hours after the cessation of respiration. In the cases of Dr. Dowler, percussion excited contractions "many hours — one to fourteen — after death, and even hours after complete dissection of the brain, spinal cord, viscera and limbs, and the removal of many pounds of blood."

We shall not on this occasion follow the writer in his general consideration of the phenomena which his experiments disclosed, and the discussion of their relations with the doctrines of Bell, Hall and others. In this portion of the article his remarks are extremely discursive, and lack that methodical connection which is desirable in the discussion of a scientific subject. The composition, too, if we may be allowed the criticism, is not always in keeping with the nature of the discussion. The writer indulges in a pertness of style which does not seem to us in good taste. He does not offer any rationale of the phenomena beyond the supposition of a peculiar property inherent in the muscular tissue, known to physiologists as the property of contractility or irritability. But this property his observations demonstrate, as he thinks, to reside solely in the muscular tissue itself, irrespective of the medulla spinalis, or any portion of the nervous system, except the filaments which may be directly interwoven with the muscular fibres. We are free to confess that the fact that contractility, in the cadaver at least, is inherent in the muscle, independently of all its nervous connections, is abundantly proven, admitting the experiments to be valid, which we have no reason to doubt. The muscular contractions must have been produced exclusively by the act of percussion upon the muscles, not involving the nerves leading to them; for the contractions were in all instances limited to the muscle or muscles struck, and, hence, by percussing the appropriate muscles, each, or all the muscles of the forearm, for example, could be produced at pleasure—supination, pronation, &c. And, again, the following consideration places the supposition that the effects were produced by the action of the blows on the nerves, and not on the muscles, out of the question:

"In striking the biceps muscle, in the manner I have described, nearly all the great nervous cords distributed to the arm, fore-arm, wrist, hand, and fingers, are equally the recipients of the percussing force, as the external cutaneus, the median, etc. Now, the same force that is applied to the nervous twigs of the biceps, ought to communicate motion to all the parts upon which the great nerves ramify; that is, to the fore-arms and fingers. This never happens in the slightest degree; the motion is limited to the muscle struck, and not transmitted to the muscles to which the nerves are distributed."

We are not, however, prepared, to admit that the reflex theory is thereby disproved. It is disproved in the instances of the experiments, but it does not follow that the muscular system during life may not sustain relations with the spinal cord through an excito-motary system, according to the doctrines of Hall and others, In short, all the facts embraced in the reflex theory may be true in the living organism, and to a certain extent in the cadaver, and, nevertheless, the muscles possess that independent contractile power which causes them to reactupon direct percussion. It is certainly true that the voluntary muscles contract in obedience to some influence emanating from the sensorium and transmitted through the nerves leading to the muscles. This influence, which we call *the will*, whatever it be, is as certainly widely different from a blow by a stick or hatchet made directly over the muscle. We should not infer that because the latter will after death occasion motions, that, therefore, the former, operating at the roots of the nerves, does not, in the living body, also occasion contractions. We know both facts to be true. In the same manner, admitting fully Dr. Dowler's experiments, we do not see that they nullify the facts belonging to the reflex theory. Each class of facts may alike be true. The force of the percussion develops the contraction after death; but what occasions the involuntary contractions of the same muscles during life, in tetanus spasms, &c? There is no direct, appreciable exciting force, in these cases, in operation. Why may not the morbid agencies lie at the nervous roots, as in the case with the normal influence derived from volition? Dr. D. deduces from his observations that the diseases just mentioned, and others, are due to the diseased condition of the muscles themselves, irrespective of any reflex current, or a centric morbid agency. This does not appear to us a logical inference. In his experiments there was a direct, palpable, powerful cause acting upon the muscular tissue itself. In spasmodic affections there is no such cause appreciable. The analogy between the two cases does not hold good. But, on the other hand, various considerations might be mentioned to sustain the theory that these effects are produced by a morbid centric cause. One of these is the fact that such centric causes are oft-times revealed by examinations after death. Another is, that spasmodic affections generally embrace a large number of muscles, and that a single muscle very seldom takes on spasmodic contraction. The latter would be oftener true if these diseases depended chiefly on the condition of the muscle itself.

We throw out these reflections as they occur to us at the moment, without taking time and space to devote to the subject that reflection which it merits, and submitting an elaborate criticism. Our readers will, of course, take them for what they are worth. We confess that we should feel great reluctance in relinquishing the doctrines of the excito-motary system, rendering intelligible, as they do, a host of pathological phenomena, which were formerly but little understood, and often-times badly treated. At the same time, there is reason to suppose that these doctrines may have been pushed too far. This is usually the case with new views after they once obtain currency. The observations of Dr. Dowler demonstrate that muscular contractility exists in the tissue, *per se*, and does not require for its exercise that the influence of the exciting cause should, in all cases, be reflected through the medulla spinalis. This fact is to be borne in mind, and it remains for future researches to determine with precision, which of the various pathological phenomena manifested in the muscular system are due respectively to a reflex-action, or to the condition of the muscular system itself.

In conclusion we give some farther quotations from the interesting article under review. The first relates to the development of *muscular contraction after death unconnected with any appreciable cause or excitant.* The reader will perhaps think, with ourselves, that the following cases tend quite as much to illustrate the functions of the nervous as the muscular system. They are certainly very curious:

"A young lady, who had been my patient, died of cholera, in 1833, at Wheeling, Virginia, during my visit to that town. I obtained, with difficulty, permission to make a *post-mortem* examination, at about three hours after death, when I found a brisk twitching of the fingers, somewhat like *subsultus tendinum*, being chiefly in the flexor muscles, which latter had suffered before death from cramps and strong flexions. Here the disease of the muscle, so to speak, was still operating!

The next case, still more remarkable, was most carefully noted at the instant of its occurrence, as follows:—1843, October 8th, 11 A. M. Peter Duffy, Irishman, aged 28, last from St. Louis, resident in New Orleans one month; sick four days; hand 23°; bend of the arm, 26°; axilla, 28° of Reaumur; body curved to the right; fists clenched; fore-arms flexed, pressing against the chest; insensible; a column of black vomit, about twenty ounces, was projected from the mouth, like a *jet d'eau*, two or three feet perpendicularly: it fell back into the mouth, and on the right side of the face; the mouth being closed, two smaller streams were forcibly discharged through the nostrils upon the breast; the jaws became fixed; the lips compressed; respiration ceased. He was dead. Every part of his body was quiescent and motionless; but, soon after, he performed the following motions, occupying, by estimation, from three to five minutes:—First he carried his left hand, by a regular motion, to his throat, then to the crown of his head; the right arm followed the same route on the right side; the left hand was then carried back to the throat; thence to the breast, reversing all its original motions; the right hand and arm performed exactly the same motions: in all, eight consecutive, identical, equable motions, in apparently equal times, and with a regularity indicative of volition, though before death he was totally insensible."

The following quotation is highly interesting:

"Post-mortem contractility, in its legal applications, is not without interest, showing, as it does, the absurdity of some grave *judicial decisions*, based solely on muscular motion, which latter has been held to be a sufficient proof of life in a new-born child, enabling the husband to inherit the estate of his wife during his lifetime. In a case given by Dr. Dunglison (*Phys.* v. i.), the Court of Exchequer (in England) and the jury decided that the child was born alive, because, when it was immersed in a warm bath, a *twitching* and *tremulous motion* of the lips appeared twice. What would the Cokes and Blackstones of the law say, agreeably to this principle, on seeing a dissected, eviscerated man performing regular flexions, extensions, pronations, supinations,—lifting heavy weights from the floor and placing them on his chest? To designate the precise moment

NOTE.—Since writing this review we have been informed by R. W. Haskins Esq. of this city, (a gentleman well known for his general scientific attainments) that spontaneous spasmodic action of the muscles after death was observed by him in several instances during the prevalence of the cholera in this place, in 1832. Mr. Haskins was at that time a member of the board of health, and was distinguished for his personal exertions in the performance of his official duties. These frequently brought him into contact with subjects recently dead with cholera. He related to us one case in which were manifest flexion and extension of the toes, frequently repeated, after the body had been laid out for burial. In another case, the muscles of the face underwent a series of contractions, producing a variety of grimaces. And, in another case, this was observed on one side of the median line, the muscles on the other side remaining quiescent. In grasping the thighs of subjects, Mr. H. frequently felt the muscles contract distinctly and forcibly, but not to produce motion of the member. Mr. H. had not supposed these phenomena were novel. They may not be so to many members of the profession, but we confess they are so to us.

when living becomes dead matter is indeed a desideratum: "to be, or not to be, that is the question" which science, humanity, and the strongest of the human passions have not been able to solve.

"Although three centuries have rolled away, no anatomist, no medical man, can think of the melacholy fate of the illustrious 'Vesalius' without emotion:

"'Vesalius' says Mr. Hallam, in his History of the Literature of Europe, " left every anatomist far behind. He was to anatomy what Copernicus was to astronomy. A superstitious prejudice against human dissections had restrained ancient anatomists in general to pigs and apes. Vesalius and students prowled by night in charnel houses, they dug up the dead, they climbed the gibbet, in fear and in silence, to steal the mouldering carcase of the murderer, the risks of ignominious punishment, and the secret stings of superstitious remorse, exalting, no doubt, the delight of those useful but not very enviable pursuits. Being absurdly accused of having dissected a Spanish gentleman *before he was dead*, Vesalius only escaped capital punishment, at the instance of the Inquisition, by undertaking a pilgrimage to Jerusalem, during which he was shipwrecked, and died of famine in one of the Greek Islands" (in the year 1563). The only proof against Vesalius was, that the young nobleman's heart *moved* (some say palpitated) when it was exposed to view by dissection- This motion was doubtless nothing more than some contractions of the muscular fibres excited by the knife (as already explained), and which may be produced in every heart recently dead, without, perhaps, a single exception. Having digressed so far, I will add that, some years since, several medical students declared to me that, while dragging an extremely large, yellow fever subject from one room to another, preparatory to dissection, the *cadaver* groaned distinctly. I arrived some time after—I found the contractile function very strong. Venesection discharged blood with force upward, against the principles of gravity, and in great quantity—(a curious subject, upon which I have the materials for a monograph). The circumstance alluded to appeared wholly inexplicable, until, at length, I heard a subject groan with my own ears,—since which I have caused many groans by placing the foot against the chest, and making strong pressure, which causes the air to be expelled through the larynx and organs of speech with noises sometimes not unlike difficult breathing and inarticulate moaning. The elasticity of the ribs causes them to return to their former states, as soon as the pressure is withdrawn; the air at the same time rushes through the opening of the glottis to fill up the vacuum in the chest.

In most biographies of Vesalius, it is said that after he had dissected down to the heart, this organ was found palpitating with life! This nobleman, be it observed, had been the patient of Vesalius, had died under his eyes, and *showed no signs of life during the early part of the dissection*, and before the exposure of the heart—the friends of the deceased had requested the post-mortem examination, and did not, it may be inferred, proceed prematurely. The operator had long before published the greatest work on Anatomy that the world had ever seen (*De Corporis Humani Fabrica*), and must have known what were the signs of death. From all these circumstances it may be inferred that the accusation upon which the Inquisition condemned him to the most cruel tortures and death, and from which Charles V saved him, was absurd in the highest degree, and originated in that post-mortem contraction above described*

The tales now circulated, chiefly by some European writers, going to show that not a few persons have been buried alive, or have shown signs of life after the phenomena supposed to belong to real death had taken place, may have no better foundation in most cases than that upon which Vesalius was exiled. It is, moreover, to be observed that most great writers upon this subject, and upon premature interment, seem to get their facts from reports and rumors not properly authenticated. Even M. Julia de Gontenelle, who has written eloquently upon this subject, seems not to have seen any of the cases which he has collected from all points of the compass, chiefly from the newspapers. Such facts afford at best

"A most lame and impotent conclusion,"

which must be productive of evil only, by arraying the strongest prejudice against the progress of science and the increase of human happiness."

In closing this review we should do injustice to our appreciation of the labors of Dr. D. if we omitted to express the great gratification with which we have read his valuable paper. His observations upon muscular contractility, and the temperature of the body after death, are of a striking character, and cannot fail to excite the lively interest of physiologists. We are sorry to learn that not only have attempts been made to deprive him of his claims to originality, but that the exactitude of his observations has been flippantly discredited. The experiments can easily be verified, which does not appear to have been done before calling his facts in question. On this subject we will say more hereafter. We trust he will be encouraged to continue and extend his experiments, relying, as he certainly may do with confidence, that justice will be done both to himself, and to the facts which he may develop.

